# *catena*-Poly[[tetra­kis­(hexa­methyl­phospho­ramide-κ*O*)bis­(nitrato-κ^2^ *O*,*O*′)dysprosium(III)] [silver(I)-di-μ-sulfido-tungstate(VI)-di-*μ*-sulfido]]

**DOI:** 10.1107/S1600536810029235

**Published:** 2010-09-04

**Authors:** Hongyang Wei, Jinfang Zhang, Chi Zhang

**Affiliations:** a Institute of Molecular Engineering and Advanced Materials, School of Chemical Engineering, Nanjing University of Science and Technology, 200 Xiaolingwei, Nanjing 210094, Jiangsu, People’s Republic of China; b Institute of Science and Technology, Jiangsu University, 301 Xuefu Road, Zhenjiang 212013, People’s Republic of China

## Abstract

Hexa­methyl­phospho­ramide (hmp), tetra­thio­tungstate, silver sulfide and dysprosium nitrate were self-assembled, forming an anionic [AgWS_4_]*_n_^n^* ^−^ chain in the title compound, {[Dy(NO_3_)_2_(C_6_H_18_N_3_OP)_4_][AgWS_4_]}_*n*_. The central Dy atom in the cation is coordinated by eight O atoms from two didentate nitrate and four hmp ligands, giving rise to a distorted square anti­prismatic structure. Together with the two nitrate ligands, the cation is univalent, which leads to the anionic chain having a [WS_4_Ag] repeat unit. The polymeric anionic chain, with W—Ag—W and Ag—W—Ag angles 161.16 (2) and 153.606 (11)°, respectively, presents a distorted linear configuration. The title compound is isotypic with other rare earth complexes.

## Related literature

For one-dimensional Mo(W)/S/Ag anionic polymers, see: Niu *et al.* (2004 ▶). For their unique properties, see: Zhang *et al.* (2007*a* ▶). For the structures of isotypic compounds, see: Cao *et al.* 2007 ▶) for Yb; Zhang *et al.* (2007**b* ▶,c* ▶) for Y and Eu; Tang *et al.* (2008*a* ▶,*b* ▶) for Nd and La.
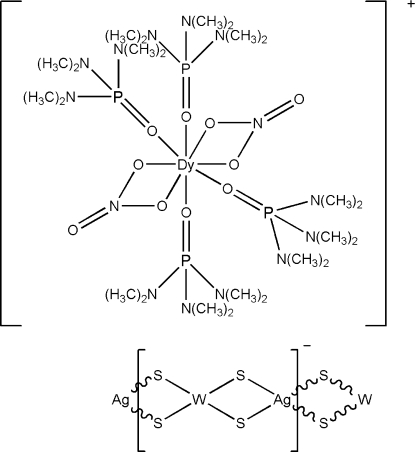


## Experimental

### 

#### Crystal data

[Dy(NO_3_)_2_(C_6_H_18_N_3_OP)_4_][AgWS_4_]
*M* *_r_* = 1423.33
Monoclinic, 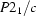

*a* = 15.790 (3) Å
*b* = 29.659 (6) Å
*c* = 11.376 (2) Å
β = 90.94 (3)°
*V* = 5326.8 (18) Å^3^
*Z* = 4
Mo *K*α radiation
μ = 4.24 mm^−1^
*T* = 153 K
0.25 × 0.2 × 0.15 mm

#### Data collection

Rigaku Saturn724+ diffractometer
Absorption correction: multi-scan (*CrystalClear*; Rigaku, 2007 ▶) *T*_min_ = 0.376, *T*_max_ = 0.529
24513 measured reflections
9675 independent reflections
8851 reflections with *I* > 2.0σ(*I*)
*R*_int_ = 0.029

#### Refinement

*R*[*F* ^2^ > 2σ(*F* ^2^)] = 0.035
*wR*(*F* ^2^) = 0.081
*S* = 1.08
9675 reflections
532 parameters
H-atom parameters constrained
Δρ_max_ = 1.12 e Å^−3^
Δρ_min_ = −0.87 e Å^−3^

### 

Data collection: *CrystalClear* (Rigaku, 2007 ▶); cell refinement: *CrystalClear*; data reduction: *CrystalClear*; program(s) used to solve structure: *SHELXS97* (Sheldrick, 2008 ▶); program(s) used to refine structure: *SHELXL97* (Sheldrick, 2008 ▶); molecular graphics: *SHELXTL* (Sheldrick, 2008 ▶); software used to prepare material for publication: *SHELXTL*.

## Supplementary Material

Crystal structure: contains datablocks I, global. DOI: 10.1107/S1600536810029235/pv2306sup1.cif

Structure factors: contains datablocks I. DOI: 10.1107/S1600536810029235/pv2306Isup2.hkl

Additional supplementary materials:  crystallographic information; 3D view; checkCIF report

## Figures and Tables

**Fig. 1.**
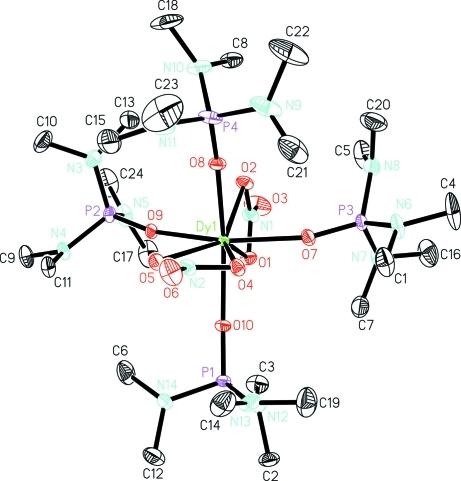
The molecular structure of the cation in the title compound, with atomic labels and 30% probability displacement ellipsoids; H atoms have been omitted for clarity.

**Fig. 2.**
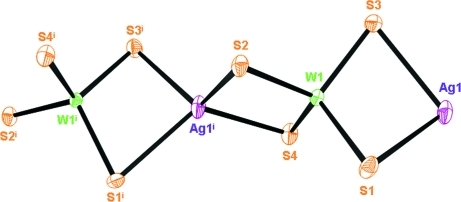
The molecular structure of a portion of the anionic chain in the title compound, with atomic labels and 30% probability displacement ellipsoids, Symmetry code: (i) x, 1/2 - y, -1/2 + z.

## supplementary crystallographic information

### Comment

One-dimensional Mo(W)/S/Ag anionic polymers have attracted much attention for their configurational isomerism (Niu *et al.*, 2004) and unique properties as functional materials, such as third-order nonlinear optical (NLO) materials (Zhang *et al.*, 2007*a*). Different solvent-coordinated rare-earth cations proved effective to obtain various configurations of anionic chains (Niu *et al.*, 2004). The title compound, {n[Dy(hmp)_4_(NO_3_)_2_][WS_4_Ag]_n_} (hmp = hexamethylphosphoramide) with a wave-like anionic chain was prepared by following such route using Dy(III)-hmp complex as counterion.

The title complex is isostructural with Yb (Cao *et al.* 2007), Y (Zhang *et al.* 2007*b*), Eu (Zhang *et al.* 2007*c*), Nd (Tang *et al.* 2008*a*), and La (Tang *et al.* 2008*b*) isomorphs. Dy^3-^ in the cation of the title complex is coordinated by eight O atoms from two nitrate and four hmp ligands. Parts of dimethylamine groups from hmp ligands have large librations reflecting a small degree of thermal disorder. In possession of two nitrate ligands, the cation in the title compound is univalent (Fig. 1), which leads to an anionic chain with a univalent repeat unit. The anionic chain in the title compound (Fig. 2) has a distorted linear configuration with W—Ag—W and Ag—W—Ag angles of 161.16 (2) and 153.606 (11) °, respectively, as reported in the other isostructural complexes quoted above.

### Experimental

Ag_2_S (1 mmol) was added to a solution of [NH_4_]_2_WS_4_ (2 mmol in 30 mL hmp) with thorough stirring for 6 h. The solution underwent an additional stirring for two minute after Dy(NO_3_)_3_.6H_2_O (1 mmol) was added. After filtration the orange-red filtrate was carefully laid on the surface with 30 ml *i*-PrOH. Orange-red block crystals were obtained after ten days.

### Refinement

H atoms were positioned geometrically and refined with riding model, with C—H = 0.96 Å and *U*_iso_ = 1.5*U*_eq_(C). The final difference map had a residual electron density in the close proximity of W1 (1.0 Å).

### Crystal data

**Table:**

[Dy(NO_3_)_2_(C_6_H_18_N_3_OP)_4_][AgWS_4_]	*F*(000) = 2820.0
*M**_r_* = 1423.33	*D*_x_ = 1.775 Mg m^−^^3^
Monoclinic, *P*2_1_/*c*	Mo *K*α radiation, λ = 0.71073 Å
Hall symbol: -P 2ybc	Cell parameters from 22684 reflections
*a* = 15.790 (3) Å	θ = 3.1–29.1°
*b* = 29.659 (6) Å	µ = 4.24 mm^−^^1^
*c* = 11.376 (2) Å	*T* = 153 K
β = 90.94 (3)°	Block, orange-red
*V* = 5326.8 (18) Å^3^	0.25 × 0.2 × 0.15 mm
*Z* = 4	

### Data collection

**Table:**

Rigaku Saturn724+ diffractometer	9675 independent reflections
Radiation source: fine-focus sealed tube	8851 reflections with *I* > 2.0σ(*I*)
graphite	*R*_int_ = 0.029
dtprofit.ref scans	θ_max_ = 25.4°, θ_min_ = 3.2°
Absorption correction: multi-scan (*CrystalClear*; Rigaku, 2008)	*h* = −19→13
*T*_min_ = 0.376, *T*_max_ = 0.529	*k* = −35→33
24513 measured reflections	*l* = −13→11

### Refinement

**Table:**

Refinement on *F*^2^	Primary atom site location: structure-invariant direct methods
Least-squares matrix: full	Secondary atom site location: difference Fourier map
*R*[*F*^2^ > 2σ(*F*^2^)] = 0.035	Hydrogen site location: inferred from neighbouring sites
*wR*(*F*^2^) = 0.081	H-atom parameters constrained
*S* = 1.08	*w* = 1/[σ^2^(*F*_o_^2^) + (0.0212*P*)^2^ + 31.1497*P*] where *P* = (*F*_o_^2^ + 2*F*_c_^2^)/3
9675 reflections	(Δ/σ)_max_ = 0.002
532 parameters	Δρ_max_ = 1.12 e Å^−^^3^
0 restraints	Δρ_min_ = −0.87 e Å^−^^3^

### Special details

**Table:**

Geometry. All e.s.d.'s (except the e.s.d. in the dihedral angle between two l.s. planes) are estimated using the full covariance matrix. The cell e.s.d.'s are taken into account individually in the estimation of e.s.d.'s in distances, angles and torsion angles; correlations between e.s.d.'s in cell parameters are only used when they are defined by crystal symmetry. An approximate (isotropic) treatment of cell e.s.d.'s is used for estimating e.s.d.'s involving l.s. planes.
Refinement. Refinement of *F*^2^ against ALL reflections. The weighted *R*-factor *wR* and goodness of fit *S* are based on *F*^2^, conventional *R*-factors *R* are based on *F*, with *F* set to zero for negative *F*^2^. The threshold expression of *F*^2^ > σ(*F*^2^) is used only for calculating *R*-factors(gt) *etc*. and is not relevant to the choice of reflections for refinement. *R*-factors based on *F*^2^ are statistically about twice as large as those based on *F*, and *R*- factors based on ALL data will be even larger.

### Fractional atomic coordinates and isotropic or equivalent isotropic displacement parameters (Å^2^)

**Table:**

	*x*	*y*	*z*	*U*_iso_*/*U*_eq_	
Dy1	0.737898 (16)	0.082563 (8)	0.82753 (2)	0.01918 (7)	
P1	0.69823 (11)	−0.03058 (5)	0.69847 (15)	0.0279 (4)	
P2	0.52175 (10)	0.13308 (5)	0.82312 (14)	0.0264 (3)	
P3	0.95850 (10)	0.09625 (5)	0.73112 (15)	0.0270 (3)	
P4	0.79491 (13)	0.14651 (6)	1.09706 (16)	0.0374 (4)	
O1	0.7239 (3)	0.10325 (13)	0.6185 (4)	0.0314 (10)	
O2	0.7515 (3)	0.15839 (13)	0.7372 (4)	0.0330 (10)	
O3	0.7248 (4)	0.17207 (18)	0.5529 (5)	0.0659 (17)	
O4	0.8012 (3)	0.02649 (14)	0.9650 (4)	0.0296 (10)	
O5	0.6686 (3)	0.04061 (14)	0.9886 (4)	0.0290 (9)	
O6	0.7373 (3)	0.00133 (18)	1.1211 (5)	0.0513 (14)	
O7	0.8757 (3)	0.08065 (13)	0.7803 (4)	0.0299 (10)	
O8	0.7725 (3)	0.12697 (14)	0.9816 (4)	0.0302 (10)	
O9	0.6029 (2)	0.10699 (13)	0.8235 (4)	0.0266 (9)	
O10	0.7065 (3)	0.01790 (12)	0.7279 (4)	0.0265 (9)	
N1	0.7335 (3)	0.14548 (17)	0.6337 (5)	0.0332 (12)	
N2	0.7359 (3)	0.02203 (17)	1.0282 (5)	0.0301 (12)	
N3	0.5230 (4)	0.17275 (19)	0.9225 (6)	0.0427 (15)	
N4	0.4445 (3)	0.09787 (18)	0.8495 (5)	0.0305 (12)	
N5	0.5054 (4)	0.1574 (2)	0.6976 (5)	0.0460 (16)	
N6	1.0341 (3)	0.0709 (2)	0.8051 (5)	0.0404 (15)	
N7	0.9563 (4)	0.0862 (2)	0.5892 (5)	0.0447 (15)	
N8	0.9832 (4)	0.14921 (18)	0.7390 (6)	0.0416 (15)	
N9	0.8966 (5)	0.1385 (2)	1.1236 (7)	0.070 (2)	
N10	0.7754 (5)	0.20026 (19)	1.0959 (5)	0.0480 (17)	
N11	0.7407 (6)	0.1235 (2)	1.2007 (6)	0.067 (2)	
N12	0.7204 (4)	−0.03686 (17)	0.5586 (5)	0.0350 (13)	
N13	0.7634 (4)	−0.06550 (18)	0.7666 (5)	0.0437 (15)	
N14	0.6045 (4)	−0.0481 (2)	0.7376 (6)	0.0475 (16)	
C1	1.0210 (5)	0.0280 (3)	0.8613 (8)	0.060 (2)	
H1A	1.0724	0.0186	0.9003	0.090*	
H1B	1.0050	0.0059	0.8032	0.090*	
H1C	0.9768	0.0307	0.9179	0.090*	
C2	0.7254 (5)	−0.0827 (2)	0.5060 (7)	0.050 (2)	
H2A	0.7390	−0.0802	0.4243	0.074*	
H2B	0.6717	−0.0976	0.5134	0.074*	
H2C	0.7685	−0.0998	0.5462	0.074*	
C3	0.6974 (6)	−0.0024 (3)	0.4746 (6)	0.052 (2)	
H3A	0.7153	−0.0114	0.3978	0.078*	
H3B	0.7245	0.0254	0.4962	0.078*	
H3C	0.6371	0.0016	0.4738	0.078*	
C4	1.1232 (5)	0.0803 (4)	0.7827 (9)	0.080 (3)	
H4A	1.1582	0.0624	0.8344	0.120*	
H4B	1.1345	0.1117	0.7963	0.120*	
H4C	1.1356	0.0730	0.7026	0.120*	
C5	0.9516 (5)	0.1825 (3)	0.6534 (11)	0.082 (4)	
H5A	0.9734	0.2118	0.6736	0.124*	
H5B	0.8908	0.1832	0.6545	0.124*	
H5C	0.9699	0.1744	0.5762	0.124*	
C6	0.5348 (5)	−0.0163 (3)	0.7479 (8)	0.059 (2)	
H6A	0.4848	−0.0321	0.7714	0.089*	
H6B	0.5245	−0.0019	0.6734	0.089*	
H6C	0.5492	0.0061	0.8058	0.089*	
C7	0.9085 (5)	0.0481 (3)	0.5430 (7)	0.057 (2)	
H7A	0.9140	0.0469	0.4591	0.085*	
H7B	0.8498	0.0516	0.5621	0.085*	
H7C	0.9299	0.0207	0.5771	0.085*	
C8	0.8031 (5)	0.2273 (2)	0.9966 (6)	0.0473 (19)	
H8A	0.7871	0.2582	1.0084	0.071*	
H8B	0.7768	0.2162	0.9256	0.071*	
H8C	0.8635	0.2252	0.9904	0.071*	
C9	0.3560 (4)	0.1074 (3)	0.8127 (7)	0.0473 (19)	
H9A	0.3203	0.0829	0.8363	0.071*	
H9B	0.3529	0.1107	0.7288	0.071*	
H9C	0.3374	0.1347	0.8492	0.071*	
C10	0.4541 (5)	0.1828 (3)	1.0010 (8)	0.059 (2)	
H10A	0.4700	0.2075	1.0513	0.088*	
H10B	0.4421	0.1568	1.0480	0.088*	
H10C	0.4045	0.1909	0.9557	0.088*	
C11	0.4536 (4)	0.0646 (2)	0.9424 (6)	0.0401 (17)	
H11A	0.4029	0.0468	0.9462	0.060*	
H11B	0.4631	0.0796	1.0162	0.060*	
H11C	0.5008	0.0453	0.9262	0.060*	
C12	0.5800 (7)	−0.0956 (3)	0.7198 (9)	0.080 (3)	
H12A	0.5232	−0.1001	0.7465	0.120*	
H12B	0.6180	−0.1148	0.7635	0.120*	
H12C	0.5827	−0.1029	0.6377	0.120*	
C13	0.5903 (5)	0.2069 (3)	0.9216 (9)	0.062 (2)	
H13A	0.5823	0.2277	0.9852	0.093*	
H13B	0.5884	0.2229	0.8483	0.093*	
H13C	0.6444	0.1924	0.9309	0.093*	
C14	0.7521 (6)	−0.0815 (3)	0.8850 (8)	0.068 (3)	
H14A	0.7979	−0.1013	0.9063	0.102*	
H14B	0.6994	−0.0974	0.8896	0.102*	
H14C	0.7515	−0.0563	0.9380	0.102*	
C15	0.6502 (7)	0.1205 (3)	1.1889 (9)	0.082 (3)	
H15A	0.6276	0.1063	1.2574	0.123*	
H15B	0.6357	0.1031	1.1205	0.123*	
H15C	0.6268	0.1503	1.1810	0.123*	
C16	1.0306 (6)	0.0958 (4)	0.5173 (8)	0.076 (3)	
H16A	1.0186	0.0877	0.4371	0.115*	
H16B	1.0782	0.0787	0.5463	0.115*	
H16C	1.0436	0.1274	0.5217	0.115*	
C17	0.5188 (6)	0.1331 (4)	0.5901 (7)	0.074 (3)	
H17A	0.5059	0.1523	0.5243	0.111*	
H17B	0.4825	0.1071	0.5874	0.111*	
H17C	0.5768	0.1236	0.5868	0.111*	
C18	0.7504 (8)	0.2257 (3)	1.1997 (8)	0.091 (4)	
H18A	0.7422	0.2568	1.1788	0.137*	
H18B	0.7940	0.2234	1.2591	0.137*	
H18C	0.6985	0.2136	1.2292	0.137*	
C19	0.8530 (6)	−0.0677 (3)	0.7356 (9)	0.075 (3)	
H19A	0.8812	−0.0897	0.7842	0.113*	
H19B	0.8787	−0.0387	0.7476	0.113*	
H19C	0.8577	−0.0762	0.6545	0.113*	
C20	1.0133 (7)	0.1691 (3)	0.8496 (9)	0.088 (4)	
H20A	1.0247	0.2006	0.8380	0.133*	
H20B	1.0642	0.1541	0.8752	0.133*	
H20C	0.9707	0.1657	0.9082	0.133*	
C21	0.9359 (6)	0.0957 (3)	1.1106 (10)	0.082 (3)	
H21A	0.9949	0.0980	1.1317	0.123*	
H21B	0.9302	0.0860	1.0304	0.123*	
H21C	0.9091	0.0742	1.1610	0.123*	
C22	0.9519 (8)	0.1714 (4)	1.1835 (11)	0.105 (5)	
H22A	1.0082	0.1594	1.1904	0.158*	
H22B	0.9305	0.1776	1.2604	0.158*	
H22C	0.9530	0.1989	1.1387	0.158*	
C23	0.7820 (10)	0.1057 (4)	1.3109 (9)	0.119 (5)	
H23A	0.7396	0.0936	1.3615	0.179*	
H23B	0.8115	0.1298	1.3507	0.179*	
H23C	0.8216	0.0825	1.2912	0.179*	
C24	0.4636 (6)	0.2014 (3)	0.6817 (9)	0.077 (3)	
H24A	0.4609	0.2087	0.5995	0.116*	
H24B	0.4954	0.2241	0.7231	0.116*	
H24C	0.4073	0.2000	0.7120	0.116*	
W1	0.216043 (15)	0.227581 (7)	0.47517 (2)	0.02023 (7)	
Ag1	0.21731 (4)	0.234185 (17)	0.21464 (4)	0.03708 (13)	
S1	0.10252 (11)	0.21233 (6)	0.36887 (14)	0.0353 (4)	
S2	0.21400 (11)	0.18483 (5)	0.63413 (13)	0.0298 (3)	
S3	0.33048 (10)	0.21154 (6)	0.37526 (14)	0.0340 (4)	
S4	0.21630 (11)	0.30059 (5)	0.51519 (14)	0.0326 (4)	

### Atomic displacement parameters (Å^2^)

**Table:**

	*U*^11^	*U*^22^	*U*^33^	*U*^12^	*U*^13^	*U*^23^
Dy1	0.01654 (13)	0.01366 (12)	0.02736 (15)	−0.00042 (10)	0.00137 (10)	−0.00093 (11)
P1	0.0336 (9)	0.0183 (7)	0.0322 (9)	−0.0052 (6)	0.0095 (7)	−0.0047 (7)
P2	0.0185 (8)	0.0312 (8)	0.0296 (8)	0.0052 (6)	0.0002 (6)	0.0048 (7)
P3	0.0173 (8)	0.0245 (8)	0.0394 (9)	0.0004 (6)	0.0043 (7)	0.0078 (7)
P4	0.0558 (12)	0.0242 (8)	0.0316 (9)	−0.0101 (8)	−0.0166 (8)	0.0004 (7)
O1	0.041 (3)	0.022 (2)	0.031 (2)	0.0000 (19)	0.004 (2)	−0.0011 (19)
O2	0.038 (3)	0.020 (2)	0.041 (3)	−0.0046 (18)	−0.002 (2)	0.003 (2)
O3	0.106 (5)	0.043 (3)	0.049 (3)	−0.017 (3)	−0.012 (3)	0.025 (3)
O4	0.021 (2)	0.026 (2)	0.042 (3)	0.0001 (17)	−0.0023 (19)	0.007 (2)
O5	0.026 (2)	0.027 (2)	0.034 (2)	0.0015 (18)	−0.0001 (19)	0.0042 (19)
O6	0.051 (3)	0.056 (3)	0.047 (3)	−0.004 (3)	−0.003 (2)	0.026 (3)
O7	0.022 (2)	0.025 (2)	0.043 (3)	−0.0013 (17)	0.0023 (19)	0.0056 (19)
O8	0.030 (2)	0.026 (2)	0.035 (2)	−0.0003 (18)	−0.0073 (19)	−0.0091 (19)
O9	0.016 (2)	0.025 (2)	0.039 (2)	0.0034 (16)	0.0001 (17)	0.0007 (19)
O10	0.032 (2)	0.0165 (19)	0.031 (2)	−0.0024 (17)	0.0003 (18)	−0.0009 (18)
N1	0.037 (3)	0.025 (3)	0.038 (3)	−0.003 (2)	0.000 (2)	0.008 (3)
N2	0.031 (3)	0.024 (3)	0.036 (3)	−0.004 (2)	−0.001 (2)	0.002 (2)
N3	0.032 (3)	0.036 (3)	0.061 (4)	0.007 (3)	0.007 (3)	−0.006 (3)
N4	0.017 (3)	0.038 (3)	0.036 (3)	0.000 (2)	−0.002 (2)	0.008 (3)
N5	0.035 (3)	0.068 (4)	0.035 (3)	−0.004 (3)	−0.007 (3)	0.022 (3)
N6	0.020 (3)	0.050 (4)	0.051 (4)	−0.001 (2)	0.002 (3)	0.027 (3)
N7	0.033 (3)	0.057 (4)	0.044 (4)	−0.008 (3)	0.006 (3)	0.005 (3)
N8	0.037 (3)	0.025 (3)	0.063 (4)	−0.002 (2)	0.010 (3)	0.007 (3)
N9	0.072 (5)	0.050 (4)	0.087 (6)	−0.013 (4)	−0.049 (4)	0.001 (4)
N10	0.086 (5)	0.025 (3)	0.032 (3)	−0.008 (3)	0.004 (3)	−0.006 (3)
N11	0.110 (7)	0.049 (4)	0.043 (4)	−0.028 (4)	−0.005 (4)	0.006 (3)
N12	0.047 (4)	0.026 (3)	0.032 (3)	−0.003 (2)	0.007 (3)	−0.004 (2)
N13	0.067 (4)	0.020 (3)	0.044 (3)	0.007 (3)	0.018 (3)	0.008 (3)
N14	0.041 (4)	0.042 (3)	0.060 (4)	−0.019 (3)	0.018 (3)	−0.018 (3)
C1	0.035 (4)	0.060 (5)	0.085 (6)	0.005 (4)	0.002 (4)	0.039 (5)
C2	0.070 (6)	0.032 (4)	0.047 (4)	−0.008 (4)	0.019 (4)	−0.014 (3)
C3	0.071 (6)	0.048 (5)	0.036 (4)	−0.005 (4)	−0.003 (4)	0.001 (4)
C4	0.021 (4)	0.111 (8)	0.108 (8)	0.004 (4)	0.006 (4)	0.065 (7)
C5	0.046 (5)	0.040 (5)	0.161 (11)	0.008 (4)	0.016 (6)	0.056 (6)
C6	0.028 (4)	0.064 (5)	0.086 (7)	0.001 (4)	−0.003 (4)	0.012 (5)
C7	0.040 (5)	0.075 (6)	0.054 (5)	0.001 (4)	0.003 (4)	−0.016 (5)
C8	0.073 (6)	0.028 (4)	0.041 (4)	−0.005 (3)	0.004 (4)	−0.004 (3)
C9	0.021 (4)	0.066 (5)	0.055 (5)	−0.003 (3)	−0.002 (3)	0.010 (4)
C10	0.056 (5)	0.054 (5)	0.067 (6)	0.020 (4)	0.021 (4)	−0.006 (4)
C11	0.026 (4)	0.048 (4)	0.046 (4)	−0.001 (3)	0.007 (3)	0.008 (4)
C12	0.090 (7)	0.054 (5)	0.098 (8)	−0.039 (5)	0.045 (6)	−0.026 (5)
C13	0.053 (5)	0.038 (4)	0.096 (7)	−0.005 (4)	0.016 (5)	−0.013 (5)
C14	0.081 (7)	0.054 (5)	0.069 (6)	0.020 (5)	0.013 (5)	0.015 (5)
C15	0.105 (9)	0.072 (6)	0.071 (7)	−0.039 (6)	0.044 (6)	−0.005 (5)
C16	0.064 (6)	0.113 (8)	0.053 (5)	−0.020 (6)	0.023 (5)	0.006 (6)
C17	0.055 (6)	0.131 (9)	0.036 (5)	−0.007 (6)	−0.008 (4)	0.007 (5)
C18	0.177 (12)	0.049 (5)	0.050 (5)	−0.039 (6)	0.045 (7)	−0.030 (5)
C19	0.050 (6)	0.081 (7)	0.095 (8)	0.017 (5)	0.011 (5)	0.022 (6)
C20	0.104 (9)	0.073 (7)	0.089 (8)	−0.053 (6)	0.035 (6)	−0.030 (6)
C21	0.057 (6)	0.068 (6)	0.119 (9)	−0.006 (5)	−0.042 (6)	0.017 (6)
C22	0.117 (10)	0.085 (8)	0.113 (10)	−0.040 (7)	−0.070 (8)	0.008 (7)
C23	0.206 (16)	0.110 (10)	0.043 (6)	0.025 (10)	0.019 (8)	0.032 (6)
C24	0.058 (6)	0.074 (6)	0.100 (8)	0.014 (5)	−0.005 (5)	0.051 (6)
W1	0.02434 (13)	0.01905 (12)	0.01724 (12)	−0.00250 (9)	−0.00159 (9)	0.00094 (9)
Ag1	0.0573 (3)	0.0347 (3)	0.0192 (2)	0.0006 (2)	−0.0009 (2)	0.0018 (2)
S1	0.0298 (9)	0.0480 (10)	0.0279 (8)	−0.0136 (7)	−0.0060 (7)	0.0045 (8)
S2	0.0448 (10)	0.0215 (7)	0.0231 (8)	0.0000 (6)	0.0021 (7)	0.0063 (6)
S3	0.0306 (9)	0.0442 (9)	0.0272 (8)	0.0058 (7)	0.0018 (7)	0.0042 (7)
S4	0.0520 (11)	0.0186 (7)	0.0271 (8)	−0.0041 (7)	0.0002 (7)	0.0019 (6)

### Geometric parameters (Å, °)

**Table:**

Dy1—O7	2.250 (4)	C6—H6A	0.9600
Dy1—O9	2.252 (4)	C6—H6B	0.9600
Dy1—O8	2.253 (4)	C6—H6C	0.9600
Dy1—O10	2.278 (4)	C7—H7A	0.9600
Dy1—O1	2.463 (4)	C7—H7B	0.9600
Dy1—O2	2.483 (4)	C7—H7C	0.9600
Dy1—O5	2.484 (4)	C8—H8A	0.9600
Dy1—O4	2.482 (4)	C8—H8B	0.9600
Dy1—N1	2.888 (5)	C8—H8C	0.9600
Dy1—N2	2.905 (5)	C9—H9A	0.9600
P1—O10	1.482 (4)	C9—H9B	0.9600
P1—N14	1.637 (6)	C9—H9C	0.9600
P1—N13	1.644 (6)	C10—H10A	0.9600
P1—N12	1.645 (5)	C10—H10B	0.9600
P2—O9	1.496 (4)	C10—H10C	0.9600
P2—N5	1.617 (6)	C11—H11A	0.9600
P2—N3	1.631 (6)	C11—H11B	0.9600
P2—N4	1.637 (5)	C11—H11C	0.9600
P3—O7	1.504 (4)	C12—H12A	0.9600
P3—N8	1.620 (6)	C12—H12B	0.9600
P3—N6	1.633 (6)	C12—H12C	0.9600
P3—N7	1.642 (6)	C13—H13A	0.9600
P4—O8	1.473 (4)	C13—H13B	0.9600
P4—N11	1.619 (7)	C13—H13C	0.9600
P4—N10	1.623 (6)	C14—H14A	0.9600
P4—N9	1.647 (8)	C14—H14B	0.9600
O1—N1	1.273 (6)	C14—H14C	0.9600
O2—N1	1.265 (7)	C15—H15A	0.9600
O3—N1	1.218 (7)	C15—H15B	0.9600
O4—N2	1.274 (6)	C15—H15C	0.9600
O5—N2	1.272 (6)	C16—H16A	0.9600
O6—N2	1.222 (7)	C16—H16B	0.9600
N3—C10	1.450 (9)	C16—H16C	0.9600
N3—C13	1.469 (9)	C17—H17A	0.9600
N4—C11	1.452 (8)	C17—H17B	0.9600
N4—C9	1.480 (8)	C17—H17C	0.9600
N5—C17	1.438 (11)	C18—H18A	0.9600
N5—C24	1.471 (10)	C18—H18B	0.9600
N6—C1	1.441 (9)	C18—H18C	0.9600
N6—C4	1.462 (9)	C19—H19A	0.9600
N7—C7	1.452 (10)	C19—H19B	0.9600
N7—C16	1.469 (9)	C19—H19C	0.9600
N8—C20	1.462 (11)	C20—H20A	0.9600
N8—C5	1.469 (10)	C20—H20B	0.9600
N9—C21	1.422 (12)	C20—H20C	0.9600
N9—C22	1.469 (11)	C21—H21A	0.9600
N10—C8	1.457 (9)	C21—H21B	0.9600
N10—C18	1.461 (10)	C21—H21C	0.9600
N11—C15	1.436 (13)	C22—H22A	0.9600
N11—C23	1.500 (12)	C22—H22B	0.9600
N12—C3	1.442 (9)	C22—H22C	0.9600
N12—C2	1.487 (8)	C23—H23A	0.9600
N13—C14	1.442 (10)	C23—H23B	0.9600
N13—C19	1.465 (10)	C23—H23C	0.9600
N14—C6	1.455 (10)	C24—H24A	0.9600
N14—C12	1.476 (9)	C24—H24B	0.9600
C1—H1A	0.9600	C24—H24C	0.9600
C1—H1B	0.9600	W1—S1	2.1929 (17)
C1—H1C	0.9600	W1—S3	2.2025 (17)
C2—H2A	0.9600	W1—S2	2.2092 (15)
C2—H2B	0.9600	W1—S4	2.2125 (16)
C2—H2C	0.9600	W1—Ag1^i^	2.9506 (7)
C3—H3A	0.9600	W1—Ag1	2.9706 (7)
C3—H3B	0.9600	Ag1—S4^ii^	2.4922 (17)
C3—H3C	0.9600	Ag1—S2^ii^	2.5708 (17)
C4—H4A	0.9600	Ag1—S3	2.6222 (19)
C4—H4B	0.9600	Ag1—S1	2.6244 (19)
C4—H4C	0.9600	Ag1—W1^ii^	2.9506 (7)
C5—H5A	0.9600	S2—Ag1^i^	2.5708 (17)
C5—H5B	0.9600	S4—Ag1^i^	2.4922 (17)
C5—H5C	0.9600		
			
O7—Dy1—O9	157.26 (15)	N8—C5—H5C	109.5
O7—Dy1—O8	88.71 (16)	H5A—C5—H5C	109.5
O9—Dy1—O8	92.58 (15)	H5B—C5—H5C	109.5
O7—Dy1—O10	93.63 (15)	N14—C6—H6A	109.5
O9—Dy1—O10	93.58 (15)	N14—C6—H6B	109.5
O8—Dy1—O10	158.03 (15)	H6A—C6—H6B	109.5
O7—Dy1—O1	81.23 (15)	N14—C6—H6C	109.5
O9—Dy1—O1	80.24 (15)	H6A—C6—H6C	109.5
O8—Dy1—O1	128.50 (15)	H6B—C6—H6C	109.5
O10—Dy1—O1	73.38 (14)	N7—C7—H7A	109.5
O7—Dy1—O2	80.41 (15)	N7—C7—H7B	109.5
O9—Dy1—O2	77.81 (15)	H7A—C7—H7B	109.5
O8—Dy1—O2	76.76 (15)	N7—C7—H7C	109.5
O10—Dy1—O2	125.17 (15)	H7A—C7—H7C	109.5
O1—Dy1—O2	51.80 (14)	H7B—C7—H7C	109.5
O7—Dy1—O5	127.06 (14)	N10—C8—H8A	109.5
O9—Dy1—O5	75.40 (14)	N10—C8—H8B	109.5
O8—Dy1—O5	79.80 (15)	H8A—C8—H8B	109.5
O10—Dy1—O5	81.40 (14)	N10—C8—H8C	109.5
O1—Dy1—O5	143.50 (14)	H8A—C8—H8C	109.5
O2—Dy1—O5	143.26 (14)	H8B—C8—H8C	109.5
O7—Dy1—O4	75.68 (14)	N4—C9—H9A	109.5
O9—Dy1—O4	126.85 (14)	N4—C9—H9B	109.5
O8—Dy1—O4	79.13 (15)	H9A—C9—H9B	109.5
O10—Dy1—O4	80.31 (14)	N4—C9—H9C	109.5
O1—Dy1—O4	143.56 (14)	H9A—C9—H9C	109.5
O2—Dy1—O4	146.19 (14)	H9B—C9—H9C	109.5
O5—Dy1—O4	51.45 (13)	N3—C10—H10A	109.5
O7—Dy1—N1	81.10 (15)	N3—C10—H10B	109.5
O9—Dy1—N1	76.46 (15)	H10A—C10—H10B	109.5
O8—Dy1—N1	102.63 (16)	N3—C10—H10C	109.5
O10—Dy1—N1	99.31 (15)	H10A—C10—H10C	109.5
O1—Dy1—N1	26.00 (14)	H10B—C10—H10C	109.5
O2—Dy1—N1	25.87 (14)	N4—C11—H11A	109.5
O5—Dy1—N1	151.84 (15)	N4—C11—H11B	109.5
O4—Dy1—N1	156.68 (15)	H11A—C11—H11B	109.5
O7—Dy1—N2	101.25 (15)	N4—C11—H11C	109.5
O9—Dy1—N2	101.07 (15)	H11A—C11—H11C	109.5
O8—Dy1—N2	75.85 (15)	H11B—C11—H11C	109.5
O10—Dy1—N2	82.28 (14)	N14—C12—H12A	109.5
O1—Dy1—N2	155.65 (14)	N14—C12—H12B	109.5
O2—Dy1—N2	152.51 (15)	H12A—C12—H12B	109.5
O5—Dy1—N2	25.82 (13)	N14—C12—H12C	109.5
O4—Dy1—N2	25.85 (14)	H12A—C12—H12C	109.5
N1—Dy1—N2	177.11 (15)	H12B—C12—H12C	109.5
O10—P1—N14	108.9 (3)	N3—C13—H13A	109.5
O10—P1—N13	116.9 (3)	N3—C13—H13B	109.5
N14—P1—N13	103.5 (3)	H13A—C13—H13B	109.5
O10—P1—N12	108.0 (3)	N3—C13—H13C	109.5
N14—P1—N12	115.8 (3)	H13A—C13—H13C	109.5
N13—P1—N12	104.0 (3)	H13B—C13—H13C	109.5
O9—P2—N5	110.9 (3)	N13—C14—H14A	109.5
O9—P2—N3	111.8 (3)	N13—C14—H14B	109.5
N5—P2—N3	106.8 (3)	H14A—C14—H14B	109.5
O9—P2—N4	108.0 (3)	N13—C14—H14C	109.5
N5—P2—N4	109.8 (3)	H14A—C14—H14C	109.5
N3—P2—N4	109.5 (3)	H14B—C14—H14C	109.5
O7—P3—N8	119.2 (3)	N11—C15—H15A	109.5
O7—P3—N6	107.4 (3)	N11—C15—H15B	109.5
N8—P3—N6	104.2 (3)	H15A—C15—H15B	109.5
O7—P3—N7	107.8 (3)	N11—C15—H15C	109.5
N8—P3—N7	103.4 (3)	H15A—C15—H15C	109.5
N6—P3—N7	115.2 (3)	H15B—C15—H15C	109.5
O8—P4—N11	111.2 (3)	N7—C16—H16A	109.5
O8—P4—N10	109.6 (3)	N7—C16—H16B	109.5
N11—P4—N10	108.5 (4)	H16A—C16—H16B	109.5
O8—P4—N9	109.1 (4)	N7—C16—H16C	109.5
N11—P4—N9	109.4 (4)	H16A—C16—H16C	109.5
N10—P4—N9	109.1 (4)	H16B—C16—H16C	109.5
N1—O1—Dy1	96.0 (3)	N5—C17—H17A	109.5
N1—O2—Dy1	95.2 (3)	N5—C17—H17B	109.5
N2—O4—Dy1	96.0 (3)	H17A—C17—H17B	109.5
N2—O5—Dy1	95.9 (3)	N5—C17—H17C	109.5
P3—O7—Dy1	158.6 (3)	H17A—C17—H17C	109.5
P4—O8—Dy1	167.2 (3)	H17B—C17—H17C	109.5
P2—O9—Dy1	167.6 (3)	N10—C18—H18A	109.5
P1—O10—Dy1	161.3 (3)	N10—C18—H18B	109.5
O3—N1—O2	121.8 (5)	H18A—C18—H18B	109.5
O3—N1—O1	121.5 (6)	N10—C18—H18C	109.5
O2—N1—O1	116.7 (5)	H18A—C18—H18C	109.5
O3—N1—Dy1	174.8 (5)	H18B—C18—H18C	109.5
O2—N1—Dy1	58.9 (3)	N13—C19—H19A	109.5
O1—N1—Dy1	58.0 (3)	N13—C19—H19B	109.5
O6—N2—O4	122.4 (5)	H19A—C19—H19B	109.5
O6—N2—O5	121.8 (5)	N13—C19—H19C	109.5
O4—N2—O5	115.7 (5)	H19A—C19—H19C	109.5
O6—N2—Dy1	171.8 (4)	H19B—C19—H19C	109.5
O4—N2—Dy1	58.2 (3)	N8—C20—H20A	109.5
O5—N2—Dy1	58.2 (3)	N8—C20—H20B	109.5
C10—N3—C13	114.4 (6)	H20A—C20—H20B	109.5
C10—N3—P2	125.1 (5)	N8—C20—H20C	109.5
C13—N3—P2	119.5 (5)	H20A—C20—H20C	109.5
C11—N4—C9	114.6 (5)	H20B—C20—H20C	109.5
C11—N4—P2	120.2 (4)	N9—C21—H21A	109.5
C9—N4—P2	122.0 (5)	N9—C21—H21B	109.5
C17—N5—C24	114.3 (7)	H21A—C21—H21B	109.5
C17—N5—P2	120.3 (6)	N9—C21—H21C	109.5
C24—N5—P2	124.7 (6)	H21A—C21—H21C	109.5
C1—N6—C4	113.1 (6)	H21B—C21—H21C	109.5
C1—N6—P3	121.8 (5)	N9—C22—H22A	109.5
C4—N6—P3	121.3 (5)	N9—C22—H22B	109.5
C7—N7—C16	111.5 (7)	H22A—C22—H22B	109.5
C7—N7—P3	120.0 (5)	N9—C22—H22C	109.5
C16—N7—P3	120.5 (6)	H22A—C22—H22C	109.5
C20—N8—C5	113.6 (7)	H22B—C22—H22C	109.5
C20—N8—P3	120.9 (6)	N11—C23—H23A	109.5
C5—N8—P3	122.5 (6)	N11—C23—H23B	109.5
C21—N9—C22	112.7 (8)	H23A—C23—H23B	109.5
C21—N9—P4	122.4 (6)	N11—C23—H23C	109.5
C22—N9—P4	124.0 (8)	H23A—C23—H23C	109.5
C8—N10—C18	115.5 (6)	H23B—C23—H23C	109.5
C8—N10—P4	119.1 (5)	N5—C24—H24A	109.5
C18—N10—P4	123.6 (6)	N5—C24—H24B	109.5
C15—N11—C23	118.4 (9)	H24A—C24—H24B	109.5
C15—N11—P4	119.7 (6)	N5—C24—H24C	109.5
C23—N11—P4	121.9 (8)	H24A—C24—H24C	109.5
C3—N12—C2	113.2 (6)	H24B—C24—H24C	109.5
C3—N12—P1	120.4 (5)	S1—W1—S3	109.94 (7)
C2—N12—P1	120.4 (5)	S1—W1—S2	108.07 (6)
C14—N13—C19	110.2 (7)	S3—W1—S2	108.76 (6)
C14—N13—P1	124.2 (5)	S1—W1—S4	108.31 (7)
C19—N13—P1	120.9 (5)	S3—W1—S4	108.59 (7)
C6—N14—C12	115.7 (7)	S2—W1—S4	113.16 (6)
C6—N14—P1	120.3 (5)	S1—W1—Ag1^i^	125.56 (5)
C12—N14—P1	120.2 (5)	S3—W1—Ag1^i^	124.49 (5)
N6—C1—H1A	109.5	S2—W1—Ag1^i^	57.64 (4)
N6—C1—H1B	109.5	S4—W1—Ag1^i^	55.53 (4)
H1A—C1—H1B	109.5	S1—W1—Ag1	58.82 (5)
N6—C1—H1C	109.5	S3—W1—Ag1	58.69 (5)
H1A—C1—H1C	109.5	S2—W1—Ag1	148.75 (4)
H1B—C1—H1C	109.5	S4—W1—Ag1	98.09 (4)
N12—C2—H2A	109.5	Ag1^i^—W1—Ag1	153.606 (11)
N12—C2—H2B	109.5	S4^ii^—Ag1—S2^ii^	93.58 (5)
H2A—C2—H2B	109.5	S4^ii^—Ag1—S3	121.50 (6)
N12—C2—H2C	109.5	S2^ii^—Ag1—S3	119.81 (6)
H2A—C2—H2C	109.5	S4^ii^—Ag1—S1	120.82 (6)
H2B—C2—H2C	109.5	S2^ii^—Ag1—S1	117.31 (6)
N12—C3—H3A	109.5	S3—Ag1—S1	86.63 (5)
N12—C3—H3B	109.5	S4^ii^—Ag1—W1^ii^	47.05 (4)
H3A—C3—H3B	109.5	S2^ii^—Ag1—W1^ii^	46.54 (3)
N12—C3—H3C	109.5	S3—Ag1—W1^ii^	137.39 (4)
H3A—C3—H3C	109.5	S1—Ag1—W1^ii^	135.88 (5)
H3B—C3—H3C	109.5	S4^ii^—Ag1—W1	151.77 (4)
N6—C4—H4A	109.5	S2^ii^—Ag1—W1	114.62 (4)
N6—C4—H4B	109.5	S3—Ag1—W1	45.86 (4)
H4A—C4—H4B	109.5	S1—Ag1—W1	45.63 (4)
N6—C4—H4C	109.5	W1^ii^—Ag1—W1	161.16 (2)
H4A—C4—H4C	109.5	W1—S1—Ag1	75.55 (5)
H4B—C4—H4C	109.5	W1—S2—Ag1^i^	75.81 (5)
N8—C5—H5A	109.5	W1—S3—Ag1	75.45 (5)
N8—C5—H5B	109.5	W1—S4—Ag1^i^	77.43 (5)
H5A—C5—H5B	109.5		

Symmetry codes: (i) *x*, −*y*+1/2, *z*+1/2; (ii) *x*, −*y*+1/2, *z*−1/2.
